# Anti-hyperglycemic, lipid-lowering, and anti-obesity effects of the triterpenes α and β-amyrenones *in vivo*

**DOI:** 10.22038/AJP.2021.18076

**Published:** 2021

**Authors:** Rosilene Gomes da Silva Ferreira, Fernanda Guilhon-Simplicio, Leonard Domingo Rosales Acho, Nayana Yared Batista, Frank do Carmo Guedes-Junior, Mayla Silva Leão Ferreira, José Fernando Marques Barcellos, Valdir Florêncio Veiga-Junior, Emerson Silva Lima

**Affiliations:** 1 *Superior Normal School, Amazonas State University, Djalma Batista 2470, Chapada, 69050-010, Manaus, Manaus, AM, Brazil*; 2 *Faculty of Pharmaceutical Sciences, Federal University of Amazonas, General Rodrigo Otávio 6200, Coroado 1, 69080-900, Manaus, AM, Brazil*; 3 *Institute of Biological Sciences, Federal University of Amazonas, General Rodrigo Otávio 6200, Coroado 1, 69080-900, Manaus, AM, Brazil*; 4 *Department of Chemistry, Military Institute of Engineering, Praça General Tibúrcio 80, Urca, 22290-270, Rio de Janeiro, RJ, Brazil*

**Keywords:** Amyrenone, Obesity, Diabetes, Glycemia

## Abstract

**Objective:**

Diabetes, obesity, and their associated metabolic disorders are public health problems that require prevention and new efficient drugs for treatment. We evaluated the anti-hyperglycemic, lipid-lowering, and anti-obesity effects of semisynthetic α, β-amyrenones (ABA).

**Materials and Methods:**

BALB/c mice were used for performing an acute model of oral carbohydrate and triglyceride tolerance, and in a streptozotocin-induced diabetes model, where glycemia and body weight changes were measured during ten days. C57BL/6 strain mice were used in the diet-induced obesity model, where lipidemia and body weight were measured during four weeks, and biochemical and histological parameters were analyzed after euthanasia. The doses considered in this study were 25, 50, and 100 mg/kg of ABA, used following some criteria for each experiment.

**Results:**

ABA 25 mg/kg reduced the postprandial glycemia peak higher than acarbose 50 mg/kg (p<0.05). ABA 50 mg/kg significantly reduced glycemia in diabetic mice compared to acarbose 50 mg/kg (p<0.05). There was a reduction in the weight of the obese animals treated with ABA 25 and 50 mg/kg (p<0.05). ABA 50 mg/kg also significantly reduced lipidemia in these animals compared to orlistat 50 mg/kg.

**Conclusion:**

This study presents evidence of ABA's action in reducing postprandial glycemia and obesity in mice.

## Introduction

Hyperglycemia, dyslipidemia, and obesity currently pose serious public health problems due to their high incidence, chronic complications, and high death rates, as well as the high social and economic costs of their treatment (ADA, 2015 ▶; IDF, 2011 ▶; Queiroz et al., 2012 ▶). They are also risk factors for metabolic syndrome, a complex disorder involving alterations in arterial pressure levels, triglycerides, high-density lipoprotein (HDL) cholesterol, and fasting glycemia, amongst others (Shin et al., 2013 ▶).

Various studies have sought natural sources of molecules that might help in the management of these syndromes, with fewer toxic effects and better therapeutic schemes, given that in many cases, the patients must use hypolipidemic drug combinations throughout their lives. Plants rich in triterpenes can regulate, concomitantly, glycemia and lipid metabolism, and have a positive role to play in metabolic disorders (Nazaruk and Borzym-Kluczyk, 2014 ▶). It has also been found that a natural mixture composed of pentacyclic triterpenes α- and β-amyrin, α and β-amyrenone, and glycoside of brein/maniladiol, at concentrations of 10 μg/mL, suppresses the production of interleukin 6 (Almeida et al., 2015 ▶). Furthermore, other studies have shown that a natural isomeric mixture of α- and β-amyrins, natural precursors of the amyrenones, from *Protium heptaphyllum *Aubl. March (Burseraceae) had anti-glycemic and lipid-reducing effects in models of diabetes induced by streptozotocin and in diet-induced hyperlipidemia in rats, in a significant manner when compared with the drugs glibenclamide 10 mg/kg and fenofibrate 200 mg/kg (Santos et al., 2012 ▶).

The present study investigated whether the oxidized derivatives of α- and β-amyrins (α and β-amyrenones, respectively), possess anti-glycemic and hypolipemiant properties superior to those of their chemical precursors. Thus, we evaluated the effects of the mixture of amyrenones *in vivo* in terms of oral tolerance to carbohydrates and triglycerides, hyperglycemia in diabetic animals, and hyperlipidemia in obese animals.

## Materials and Methods

**Animals and groups**

The project was approved by the Ethics Committee on the Use of Animals of the Federal University of Amazonas (process nº 014/2014). Male mice of the C57BL/6 and BALB/c strains (approximately 18±2 g) were acquired from the São Paulo University (USP) vivarium.

C57BL/6 mice (eight weeks old) were used as subjects for hypercaloric diet-induced obesity. BALB/c (six weeks old) were used for inducing diabetes by streptozotocin and nicotinamide and for acute tests for oral triglyceride tolerance (OTTT) and oral carbohydrate tolerance (OCTT) with sugars of different complexities (maltose, sucrose, and starch). 

In all cases, the animals were acclimatized to the environment at least one week before the tests, with water and food available *ad libitum*. When necessary, the food was retired for some hours before tests. 

In this study, we employed the following codes for identifying the animal groups: GB (Basal group), in which the animals did not receive any treatment; GP (positive control), in which the experimental animals (diabetic or obese mice) received vehicle; GCP (standard group), in which the diabetic or obese mice were treated with a standard-drug of the respective test; ABA (test group), in which the animals were treated with amyrenones. A number after code identifies the dose of each group GCP or ABA. For example, ABA25 is the group treated with ABA at 25 mg/kg of bodyweight.

**Amyrenone**

The mixture of α, β-amyrenone (ABA) was obtained by oxidation of α, β-amyrin isolated from Amazonian Protium oleoresins, as previously described (Ferreira et al., 2017). For tests, ABA was suspended in 3% Kolliphor in saline solution, which is referenced as the vehicle in the methodology. 

**Toxicity analysis**

Twelve animals received the maximal dose of 300 mg/kg of bodyweight repeatedly for 12 days via oral route (gavage). We observed abnormal morphological and behavior signs of toxicity following Brazilian national protocols to study new products (ANVISA, 2004; ANIVSA, 2010). We chose spaced doses within a safe interval to use in the pharmacological tests based on these observations. 

**Effects in the oral carbohydrate tolerance test (OCTT)**

The method used for this experiment was adapted from Santos et al. (2012) ▶. We used carbohydrates maltose, sucrose, and starch as overload sugars. Arcabose (Glucobay ®BAYER) was the standard-drug of this test. A blood sample was taken from the tail of the animals in all the groups to analyze basal glycemia with an *Accu-Check *digital glucometer (Roche Diagnostics, Mannheim, Germany). An equivalent of 2 g of carbohydrate (maltose) was administered to each animal, which was divided into GB, CP, GCP, ABA25, and ABA50, each one containing six animals, after 30 min. Glucose levels were measured after 30, 60, and 90 min. The same procedure was adopted for sucrose and starch, however, only at a concentration of 50 mg/kg, owing to the number of available animals.

**Effects in the oral triglyceride tolerance test (OTTT)**

Following the methodology of Adisakwattana (2010) ▶, a volume of 200 µl of a commercial emulsion of cod liver oil (SCOTT®) was administered by gavage in male BALB/c mice. Orlistat (Xenical®) was the standard-drug of this test. We used five groups of six animals, organized into GB, CP GCP50, ABA25, ABA50, and ABA100. Blood samples were collected from the tails before ingestion (fasting) and after intervals of 30, 60, 120, and 180 min. The triglyceride content was determined using an automatic analyzer and a commercial enzymatic *Siemens* kit.

**Effects on glycemia in diabetic mice**

After a 10 hr fasting period, type II diabetes was induced in 45 male BALB/c mice by intraperitoneal administration of nicotinamide (210 mg/kg). After 20 min, an injection of streptozotocin (STZ) 180 mg/kg was given (Ramirez-Espinosa et al., 2011 ▶). Arcabose (Glucobay ®BAYER) was the standard-drug of this test. 

Glycemia in all the animals was measured by analyzing tail blood samples in an *Accu-Check* digital glucometer (Roche Diagnostics, Mannheim, Germany) 96 hr after administration of STZ. Animals with a fasting blood glucose level of 300 mg/dl were considered diabetic. Mice classified as diabetic were distributed in four groups of eight animals: CP, GCP50, ABA25, and ABA50. Animals without diabetes composed the GB group. The animals were treated for ten days. Every three days, glycemia and body weight were measured. At the end of the treatment, the animals were euthanized, and the organs were removed for histological analysis.

**Effects on hyperlipidemia and bodyweight of obese animals**

We used a total of 35 animals in this analysis. Five received a normal diet (Presence™ commercial feed), and thirty received a hypercaloric and hyperlipidemic diet (modified feed). The centesimal composition (g/100 g) of the modified feed contained carbohydrate, 67.01%; fat, 16.33%; protein, 7.36%; moisture, 7.07%, and ash, 3.23%. Orlistat (Xenical®) was the standard drug of this test.

In the fourteenth week, the animals that received the hypercaloric diet were redistributed in five new groups of six animals: GB, GP, GCP50, ABA25, and ABA50. The animals were treated for four weeks, and the quantities of food and water consumed were measured weekly. At the end of the experiment, the animals were fasted for 10 hr before blood sampling, and afterward anesthetized with ketamine and xylazine and euthanized for the removal of the visceral adipose tissue, liver, and kidneys. Blood was collected in syringes with heparin, and the plasma was used for the analysis of biochemical parameters using specific kits.

**Biochemical analyses**

The plasma levels of total cholesterol, triglycerides, glucose, urea, creatinine, aspartate aminotransferase, and alanine aminotransferase were measured using specific *Siemens* kits following the manufacturer's recommendations, using a digital biochemical analyzer (*Dade Behrering dimension RLmax*). 

**Histological analyses**

Tissue samples of the liver and kidney were fixed with 4% buffered formalin and embedded in paraffin. Sections of 5 µm were cut and stained with hematoxylin and eosin (HE), and analyzed and photographed in an optical microscope at a final magnification of 100x. Sections of adipose tissue were used to analyze abnormalities on adipocytes. For this, we used the software ImageJ (NIH, Bethesda, MD, EUA) to measure 100 adipocytes for each mice and to calculate the average cell surface area (*μ*m^2^) at the at 20x magnification. 

**Statistical analyses**

The data were developed graphically (*Interval plot* and *Boxplot*), and the mean, standard deviation, and 95% confidence level (IC95%) were calculated for data that presented a normal distribution using the Shapiro-Wilk test, and variance homogeneity, via Bartlett's test. In case of rejection of the normal hypothesis or variance homogeneity, the median was calculated. For normally distributed data with variance homogeneity, the ANOVA and Tukey tests were applied. In cases of rejection of the normal hypothesis, the Kruskal-Wallis or Wilcoxon non-parametric tests were used. A 5% significance level was used for the tests.

## Results

**Toxicity analysis**

The initial dose evaluated was 300 mg/kg. No signs of growth inhibition or serious behavioral or clinical changes were observed in most animals, although there was one death. During the experiment, a marked increase in weight was seen in all animals, without any increase in food intake. After twelve days, there was an increase in the size of the feces, but this quickly returned to normal. Alterations in color appear in the kidneys in five of the treated mice and the liver of two animals. Given these findings, this dose was considered toxic, and the doses for the biological activity trials were established well below this level (i.e. 25, 50, and 100 mg/kg), to avoid any interference in the results.

**Effects on triglyceridemia following oral hyperlipidic overloading**

**Figure 1:**
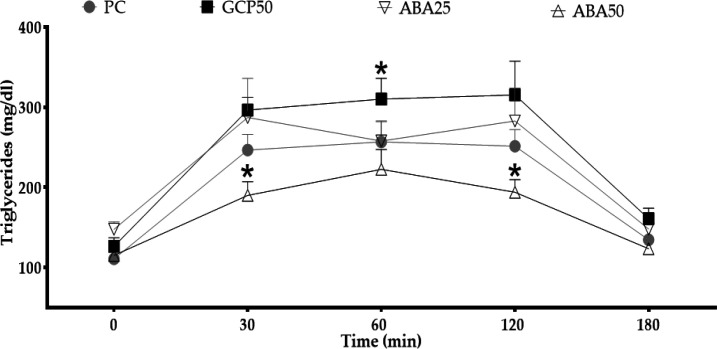
Effects of the mixture of α, β-amyrenone in the oral triglyceride tolerance test. PC, positive control; GCP50, orlistat at 50 mg/kg; ABA25 and ABA50, α, β-amyrenone at 25 or 50 mg/kg, respectively. n=6. *p<0.05 (Kruskal-Wallis Test) in comparison to the positive control

ABA reduced plasmatic levels of triglycerides at doses of 50 mg/kg, but not at 25 mg/kg. At a dose of 50 mg/kg, the reduction was significant compared to the positive control group (p0.05) measured at 30 and 120 min. At these times, plasmatic triglyceride levels in the treated animals were 189.9±38.1 and 193.9±35.4 mg/dl, respectively (Figure 1).

**Effects on the bodyweight in models of hyperlipidemia and obesity** ***in vivo***

There were no significant differences in basal weight between the animals in any group, or between groups. By the 14th week, when it was found that bodyweight in those groups fed with the modified feed was significantly greater (p<0.05) than those provided with the standard meal (35.7±2.1 g and 27.0±0.9 g respectively), treatment of the different groups began as follows: GCP25, orlistat 25 mg/kg; ABA25 and ABA50, α, β-amyrenone 25 and 50 mg/kg; and CP and GB, the vehicle only (physiological saline and Kolliphor 3%).

**Figure 2:**
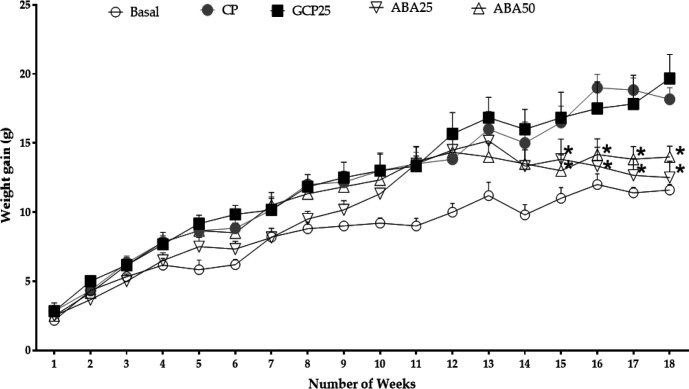
Effects of treatments α, β-amyrenone on body weight of the mice. Basal, normal feed; CP, positive control; GCP25, orlistat at 25 mg/kg; ABA25 and ABA50, α, β-amyrenone at 25 or 50 mg/kg, respectively. n=6. *p<0.05 (Kruskal-Wallis Test) in comparison to the positive control

After 18 weeks, there was a significant reduction in body weight in the ABA25 group (from 35.5±2.1 to 32. 8±2.8 g, or a 7.6% reduction), in comparison with the GCP (from 33.2±2.79 to 36.8±3.5 g, or a 9.7% increase), the CP (33.5±3.7 to 36.7±2.3 g, an 8.7% increase), and ABA50 (31.8±3.8 to 32.3±2.7 g or a 1.2% increase) groups (Figure 2).

**Histological analysis**

**Figure 3:**
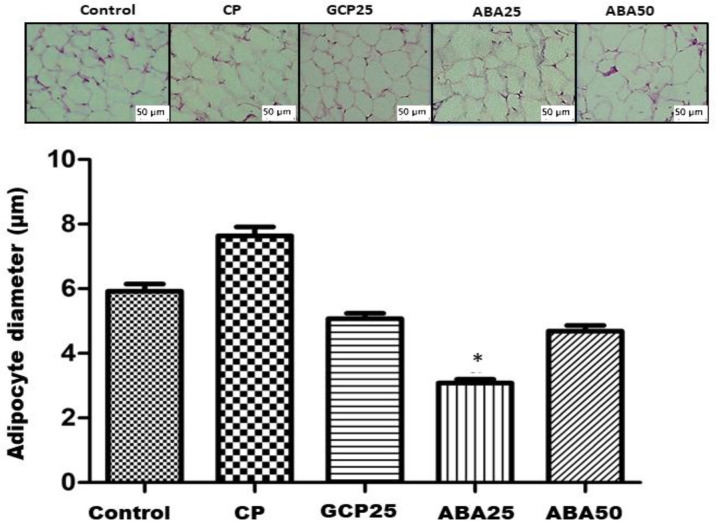
Histological sections and diameter average of adipocytes from mice. CP, positive control; GCP25, orlistat at 25 mg/kg; ABA25 and ABA50, α, β-amyrenone at 25 or 50 mg/kg, respectively. n=6. *p<0.05 (Kruskal-Wallis Test) in comparison to the positive control. Hematoxylin-eosin-stained (HE-stained) microscopic images taken at 40x magnification

The adipocytes showed a decrease in size that was directly proportional to the weight reduction of the animals, which co-occurred with the results presented in Figure 3. The adipocytes of the GCP25 group returned to the morphological pattern of the control group. The ABA25 group showed the same tendency.

These data corroborate the weight gain data presented in Figure 2. Biochemical parameters measured at the end of the experiment indicated that the induction of obesity by hypercaloric and hyperlipidemic diet produced an increase in plasmatic levels of glucose, cholesterol, and triglycerides in the different groups subject to the diet. We also observed an increase in the levels of glycemia and total cholesterol total, triglycerides, and AST, in the positive control group compared to the other groups (Table 1).

We concentrated on the liver and kidneys for the histopathological analysis since these are the organs most directly linked with the metabolism of carbohydrates and lipids, and/or the metabolism and excretion of xenobiotics. In the histopathological analysis of the liver, those from animals in GB did not present any damage. In the ABA50 group, the liver of one animal showed fatty degeneration, apoptotic cells (pyknotic nuclei), activated Kupffer cells, and some binucleate hepatocytes. 

Animals in the ABA25 group also had moderate fatty degeneration and presented apoptotic cells and binucleate hepatocytes around the centrilobular vein. In the GCP, animals treated with orlistat 25 mg/kg showed intense cytoplasmic vacuolization, steatosis, cellular degeneration, and apoptotic cells. Untreated animals showed hypercellularity in the hepatocyte cords, areas of cellular degeneration, and apoptosis. 

**Figure 4:**
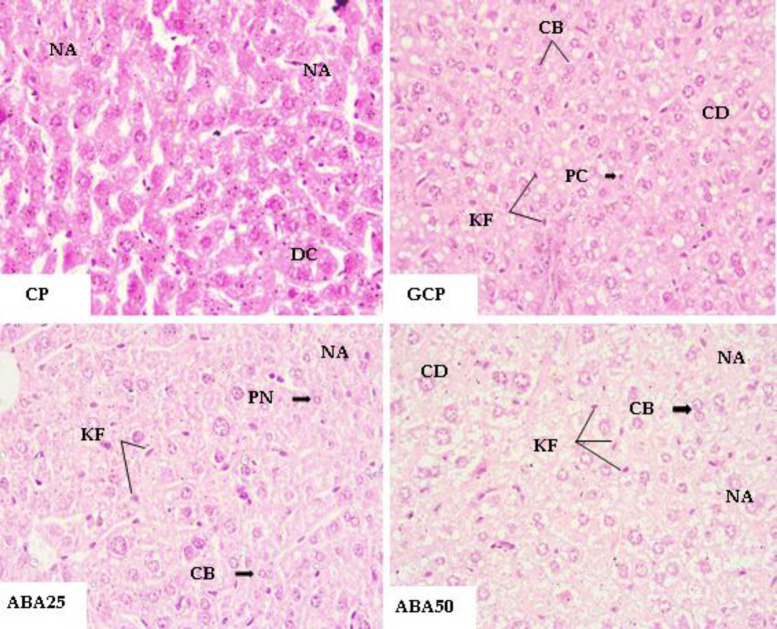
Photomicrographs of a histological section of the mouse livers. CP, positive control; GCP, Orlistate at 25 mg/kg; ABA25 and ABA50, α, β-amyrenone at 25 or 50 mg/kg, respectively. CB, Binucleate cells; CD, cellular degeneration; KF, Kupffer cells; PN, pyknotic nucleus; and NA, necrotic areas. HE-stained microscopic images were taken at 40x magnification

The incidence of binucleate hepatocytes was also high. There was no hepatic architecture alteration or intense infiltration of inflammatory cells. The majority of the altered hepatocytes presented cytoplasmatic degeneration accompanied by a change in size and shape, losing their polyhedral characteristic and often showing hypertrophy (Figure 4). 

**Table 1:** Biochemical parameters in mice subjected to a hypercaloric diet

Group	Glucose	Total cholesterol	Triglycerides	Creatinine	ALT	AST
	mean±standard deviation (mg/dL)				Q_1-_Med-Q_3_	
Basal	96.8±9.3*	83.2±4.1*	43.3±7.8*	0.50±0.3	36-57-65	67-90-124
Vehicle	128.7±4.8	115.3±13.8	87.3±23.9	0.55±0.3	23-25-33	117-230-317
ABA25	106.2±16.1*	106.7±14.9	40.3±3.8*	0.58±0.2	26-33-38	67-93-102
ABA50	106.0±7.8*	118.1±13.4	41.5±7.2*	0.85±0.3	17-19-28*	62-87-103
Orlistat	95.7±12.3*	126.5±13.5	61.3±8.9*	0.73±0.3	27-30-54	69-134-156

ALT, alanine aminotransferase; AST, aspartate aminotransferase; Q*x*, Quartis. n=6. *p<0.05 (Kruskal-Wallis Test) in comparison to the positive control (vehicle).

The kidneys of animals in the GB group presented a normal architecture of the glomeruli and intact Bowman's capsules. The renal tubules included proximal convoluted tubules composed of large pyramidal cells with brush border, and distal convoluted tubules of cube-shaped cells and more prominent luminal centers. In the CP group, with a hypercaloric and hyperlipidemic diet, kidneys presented vacuolar degeneration. The renal tubules in small areas exhibited tubular vacuolation with a loss of brush border. The Bowman's space was reduced in two animals from this group, accompanied by a cellular structure associated with glomerular and tubulointerstitial necrosis, and characterized by hydropic degeneration of the glomeruli. Animals in the GCP group presented structural histological alterations in the glomerulus and both the proximal convoluted tubules and distal convoluted tubules.

Kidneys exhibited degeneration and hypercellularity of the glomeruli. A tubulointerstitial infiltration of lymphocytes and macrophages was observed, and the renal tubules had become vacuolated and lost their brush borders. Congestion was detected near areas of necrosis. In the ABA25 group, the results showed structural histological alterations in the glomerulus and the proximal convoluted tubules and the distal convoluted tubules. In this group, we observed cellular degeneration and hypercellularity of the glomeruli and tubules. 

The renal tubules had become vacuolated with the loss of brush borders. It was possible to note congestion near areas of necrosis and a limited inflammatory infiltrates associated with necrosis (not present in all animals in this group). Eosinophilia was evident in the material that exhibited necrosis.

The kidneys of animals in the ABA50 group exhibited fewer structural histological alterations in the glomerulus and the proximal and distal convoluted tubules. The kidneys of mice in the GTA50 group showed less cellular degeneration and hypercellularity than those in the ABA25 group. The renal tubules were vacuolated, and the lumen diminished.

**Effects on the oral carbohydrate tolerance test (OCTT)**

At a dose of 50 mg/kg, ABA reduced glycemia in animals that received maltose and sucrose, but not in those that received starch (Figure 6). In animals that received maltose, the reduction was significant (p0.05, measured at 30, 60, and 90 min) compared to the positive control group. The latter exhibited glycemia of 173.40±5.1 mg/kg, 135.6±11.1 mg/dl and 128.0±10.9 mg/dl, while the ABA-treated animals exhibited glycemia of 145±33.6 mg/kg, 109±12.22 mg/dl and 105.0±7.6 mg/dl, respectively. Additionally, the reduction in glycemia was greater than that shown by acarbose at 60 min (125.8±15.0 mg/dl) and 90 min (122.2±9.3 mg/dl).

**Figure 5:**
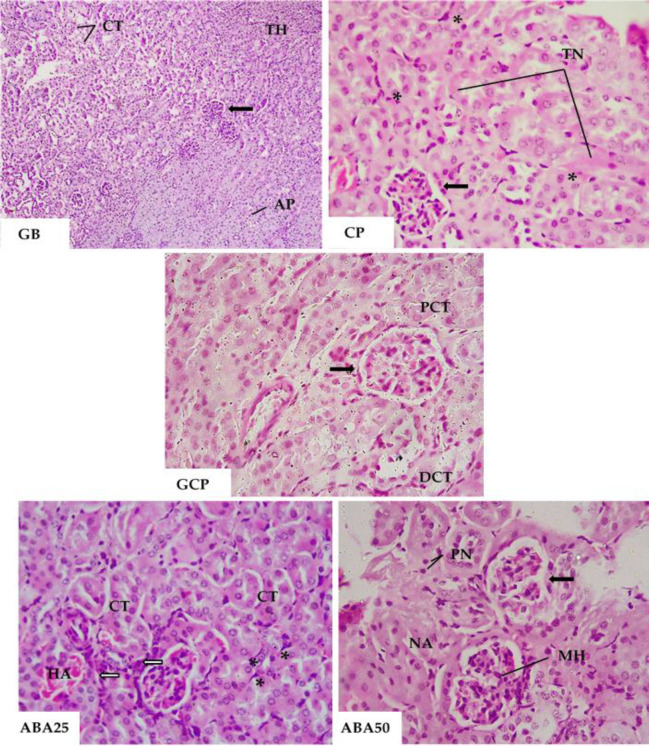
Photomicrographs of a histological section of mouse kidneys. GB, basal group; CP, positive control; GCP, orlistat at 25 mg/kg; ABA25 and ABA50, α, β-amyrenone at 25 or 50 mg/kg, respectively. AP, tubular cells in apoptosis; CT, convoluted tubules in degeneration; DCT, distal convoluted tubule; HA, hemorrhagic area; MH, mesangial hyperplasia; NA, necrotic area; PCT, normal proximal convoluted tubule; PN, pynotic nuclei (PN); TH, tubular hyperplasia; and TN, tubular necrosis. HE-stained microscopic images. GB image was taken at 10 x magnification. CP, GCP ABA25, and ABA50 images were taken at 40x magnification

**Figure 6:**
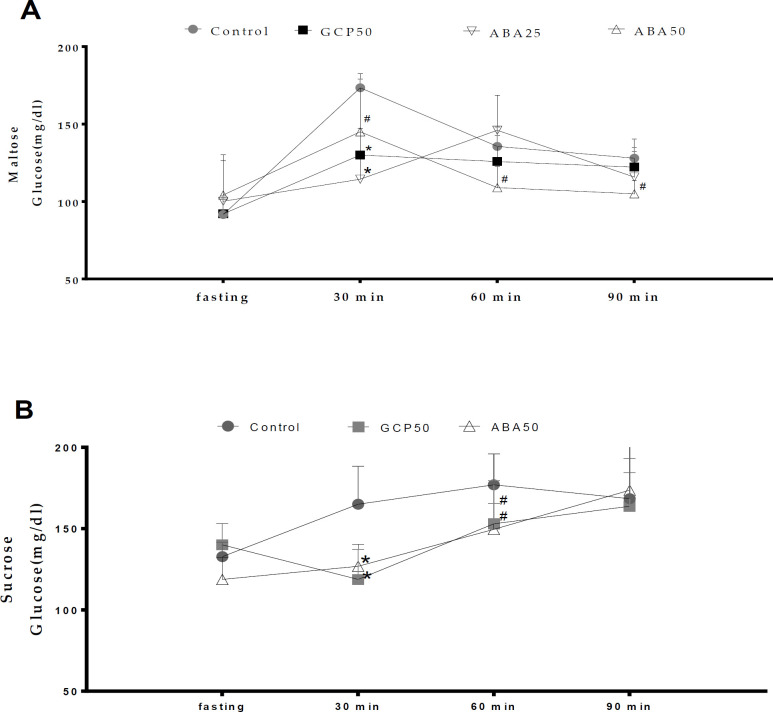
Effects of α, β-amyrenone mixture on levels of glycemia following the oral administration of maltose (A) and sucrose (B) in Balb/c mice. CP, positive control; GCP50, orlistat at 50 mg/kg; ABA25 and ABA50, α, β-amyrenone at 25 or 50 mg/kg, respectively. n=6. *p<0.01 or #p<0.05 (Kruskal-Wallis Test) in comparison to the control

In animals that received sucrose, the reduction was observed only at 30 and 60 min, when the mice exhibited glycemia of 126.8±12.2 and 149.6±14.4 mg/dl, respectively. This reduction was significant (p0.05), which presented levels of 165.0±20.9 and 168.4±28.9 mg/dl, at those time-points. However, the reduction was no greater than that exhibited with acarbose, where levels were 116.6±17.7 and 151.4±25.05 mg/dl in the first measurements. 

**Effects on glycemia** ***in vivo***

On the third day of treatment, we observed a significant increase in glycemia (p<0.05) in animals across the groups as follows: from 421.5±29.5 to 505±57.1 mg/dl in the CP group; from 416±17.7 to 458±58.0 mg/dl in the ABA25 group; and from 406.7±17.5 to 519.6±25.7 mg/dl in the ABA50 group. In contrast, there was a reduction in the GCP50 group from 449.2±59.1 to 241.2±45.9 mg/dl. 

On the sixth day, ABA at concentrations of 25 and 50 mg/kg reduced glycemia values from 458±58.0 to 140±50.6 mg/dl, and from 519.6±25.7 to 187.7±42.7 mg/dl, respectively. This was significant (p<0.05) compared to the CP group, with a reduction from 505±57.1 to 478.0±47.6 mg/dl, and with the GCP50 group, which also exhibited a reduction from 241.2±45.9 to 198.5±16.5 mg/dl.

**Figure 7:**
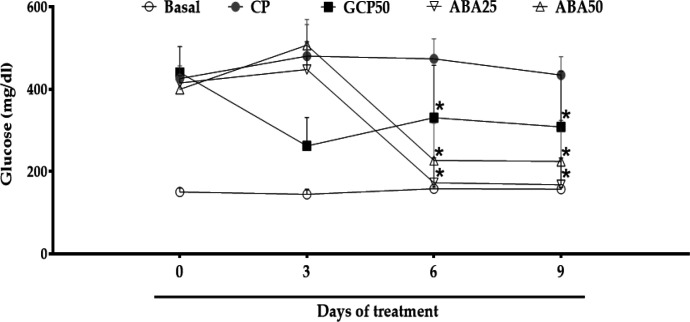
Effects of the administration of α, β-amyrenone on glycemia in mice with diabetes induced by streptozotocin+nicotinamide. CP, positive control; GCP50, orlistat at 50 mg/kg; ABA25 and ABA50, α, β-amyrenone at 25 or 50 mg/kg, respectively. n=8. *p<0.05 (Kruskal-Wallis Test) in comparison to the control (CP)

On day nine, ABA at concentrations of 25 and 50 mg/kg maintained a significant (p<0.05) reduction in glucose (from 140±50.6 to 140.25±35.2 mg/dl and from 187.7±42.7 to 187.6±44.2 mg/dl, respectively), when compared to the CP group (478.0±36.3 to 434.3±36.3 mg/dl). The GCP50 showed an increase in blood glucose from 198.5±16.5 to 257.6±88.6 mg/dl (Figure 7).

## Discussion

Studies report that the triterpene α, β-amyrin, and the resin of *Protium heptaphyllum* have a hypoglycemic and lipid-lowering action. However, these studies also acknowledge that the chemical characteristics and mechanisms are not fully understood (Santos et al., 2012 ▶). Because of this, we decided to evaluate whether ABA had similar effects. We employed a methodology identical to recent studies of the antidiabetic and anti-obesity action of different molecules, focusing on the impact of the isomers on the digestive enzymes involved in the metabolism of lipids and glycides.

This study showed that ABA in different concentrations, reduced levels of postprandial glycemia in animals submitted to the oral tolerance tests for maltose and sucrose, such as acarbose. Santos et al. (2012) ▶ also demonstrated that α, β-amyrins, from which α, β-amyrenone was derived, gave similar results in oral glucose tolerance tests realized under the same conditions. However, there was a more significant effect 60 and 90minutes following gavage with glucose.

Animals with diabetes induced by streptozotocin and nicotinamide presented a reduction in levels of plasmatic glucose following treatment with ABA when compared with those treated with acarbose. Santos et al. (2012) ▶ demonstrated that a mixture of α, β-amyrin from *P. heptaphyllum* had a hypoglycemic effect and acted as a protector for the pancreatic islets in mice at concentrations of up to 100 mg/kg when compared to the standard glibenclamide. In that case, the authors suggested that the action might have been mediated by more than one mechanism. Some studies have indicated that the triterpenes, especially the pentacyclic triterpenes, may have insulin-sensitizing properties, which might also explain the hypoglycemic effect of amyrenone (Ramirez-Espinosa et al., 2011 ▶).

ABA was able to reduce plasmatic levels of triglycerides and the animals' weight at the tested concentrations. Lipid levels were measured to define the degree of metabolic disturbance. Carvalho et al. (2015) ▶ obtained the same result with the triterpenic mixture of resin extracted with methane/dichloromethane (4:1) from *P. heptaphyllum* in a chronic test with the standard orlistat. Santos et al. (2012) ▶, using the mixture α, β-amyrin obtained from the same species, also reported positive effects concerning plasmatic levels of both cholesterol and triglycerides, at an optimum dose of 100 mg/kg, compared to the standard fenofibrate.

Obesity, taken as a multi-factor disequilibrium but essentially inflammatory, was approached by this study with the same reasoning as in Carvalho et al. (2017) ▶. In those studies, using the resin and the mixture α, β-amyrin from *P. heptaphyllum*, a reduction in weight, abdominal fat, and inflammatory markers was observed in treated mice compared to mice receiving sibutramine. There was a reduction in adipocytes and a positive modulation of the hormones leptin and ghrelin. In the current study, the mixture α, β-amyrenone at concentrations of 25 and 50 mg, reduced body weight during the four weeks of treatment compared to the standard orlistat.

In the current study, the mixture of ABA also reduced glycemic levels after carbohydrate (maltose and sucrose) loading, interfering in their digestion, affecting sugar absorption, and reducing the calories derived from carbohydrates. In this manner, it influenced the non-uptake of glucose by the adipocytes, impeding the synthesis and accumulation of triacylglycerols (Gupta et al., 2014 ▶). 

The reduction in weight may be associated with other unverified activities, such as those related to the anti-inflammatory actions already noted for other structurally similar triterpene compounds. Earlier studies with ursolic and oleanolic acids, which have the same chemical core, demonstrated a significant reduction in physical and biochemical parameters in mice subjected to a hypercaloric diet (Melo et al., 2011 ▶; Okoye et al., 2014 ▶).

The organs studied in detail were the liver and kidneys. The liver in obese mice presented biochemical parameters like those found in non-alcoholic fatty liver disease in man, which is related to inflammation and the consequent liberation of enzymes and oxidative stress, favoring hepatocytes' degeneration fibrosis, and carcinogenicity. Overall, cirrhosis has no development in these scenarios, and cirrhosis is not a prerequisite for neoplasic lesions (Nakamura and Terauchi, 2013 ▶). 

The kidneys presented vacuolar degeneration and infiltration of inflammatory cells with the hypercellularity of the glomeruli. However, these characteristics are different from those found in renal disease associated with diabetes in humans caused by mesangial proliferation. The same occurs as in the murine strain more often used for this analysis, the C57BLKS/J (Sharma et al., 2003 ▶), which does not necessarily provoke clinical manifestations. 

Another relevant consideration is that not all the strains share the same organic lesion pattern for the same disease, which leads to the need to have clear objectives for the analysis, depending on the specific animal strain selected for a given study. C57BL mice, for example, are more resistant to diabetes induced by streptozotocin and to associated renal lesions, compared to the C57BLKS/J strain that carries the mutation db/db (Sharma et al., 2003 ▶). Because of technical difficulties in obtaining animals for the experiments, it was decided to use the same strain for tests with the mixture and the histological analysis.

The ABA had an anti-hyperglycemic action in the oral carbohydrate tolerance test when using maltose and sucrose. However, it did not demonstrate any action when starch was used to induce glycemia. The mixture reduced plasmatic levels of triglycerides in animals subject to the oral lipidic emulsion markedly more than the standard orlistat. There was a significant reduction in the body weights of obese mice treated with ABA after four weeks of treatment, which indicates a possible anti-obesity effect. The mixture did not cause evident toxicity in terms of alanine aminotransferase enzymatic and aspartate aminotransferase activity. This fact is because alterations in the liver and kidneys that can be provoked by the hypercaloric diet itself, as suggested by the histological patterns of lesions, were attenuated by the treatment. 

The low solubility of ABA and animals' death during the induction of obesity and diabetes have imposed challenges in this study's conduction. Despite that, our results suggest that ABA is promising for further complementary studies involving disturbances in lipids and carbohydrates' metabolism. It is interesting to investigate the molecular mechanisms of their hypoglycemic, lipid-reducing, and anti-obesity actions since it is a low-cost molecule with expressive pharmacological potential. 

## References

[B1] Adisakwattana S (2010). Lipid-lowering mechanisms of grape seed extract (Vitis vinifera L) and its antihyperlipidemic activity. J Med Plant Res.

[B2] Almeida PD, Boleti AP, Rudiger AL, Lourenco GA, Veiga-Junior VF, Lima ES (2015). Anti-inflammatory activity of triterpenes isolated from Protium paniculatum oil-resins. Evid Based Complement Alternat Med.

[B3] American Diabetes Association (ADA) (2015). Standards of medical care in Diabetes - 2015: Summary of revisions. Diabetes Care.

[B4] Brazilian National Health Surveillance Agency (ANVISA) (2004). Guia para realização de estudos de toxicidade pré-clínica de fitoterápicos.

[B5] Brazilian National Health Surveillance Agency (ANVISA) (2010). Guia para a condução de estudos não clínicos de segurança necessários ao desenvolvimento de medicamentos.

[B6] Carvalho KM, Marinho-Filho JD, Melo TS, Araujo AJ, Quetz JS, Cunha MP, Melo KM, Silva AA, Tome AR, Havt A, Fonseca SG, Brito GA, Chaves MH, Rao VS, Santos FA (2015). The resin from Protium heptaphyllum prevents high-fat diet-induced obesity in mice: Scientific evidence and potential mechanisms. Evid Based Complement Alternat Med.

[B7] Carvalho KM, Melo TS, Melo KM, Quinderé AL, Oliveira FT, Viana AF, Nunes PI, Quetz JD, Viana DA, Silva AA, Havt A, Fonseca SG, Chaves MH, Rao VS, Santos FA (2017). Amyrins from Protium heptaphyllum reduce high-fat diet-induced obesity in mice via modulation of enzymatic, hormonal, and inflammatory responses. Planta Med.

[B8] Gupta M, Sharma P, Nath AK (2014). Purification of a novel alpha-amylase inhibitor from local Himalayan bean (Phaseolus vulgaris) seeds with activity towards bruchid pests and human salivary amylase. J Food Sci Technol.

[B9] International Diabetes Federation (IDF) (2013). Update of mortality attributable to diabetes for the IDF diabetes atlas: Estimates for the year 2011. Diabetes Res Clin Pract.

[B10] Queiroz MS, Janebro DI, Cunha MA, Medeiros JS, Sabaa-Srur AU, Diniz MF, Santos, SC (2012). Effect of the yellow passion fruit peel flour (Passiflora edulis flavicarpa deg) in insulin sensitivity in type 2 diabetes mellitus patients. Nutr J.

[B11] Melo CM, Morais TC, Tome AR, Brito GA, Chaves MH, Rao VS, Santos FA (2011). Anti-inflammatory effect of alpha, beta-amyrin, a triterpene from Protium heptaphyllu, on cerulein-induced acute pancreatitis in mice. Inflamm Res.

[B12] Nazaruk J, Borzym-Kluczyk M (2014). The role of triterpenes in the management of Diabetes mellitus and its complications. Phytochem Rev.

[B13] Nakamura A, Terauchi Y (2013). Lessons from mouse models of high-fat diet-induced NAFLD. Int J Mol Sci.

[B14] Okoye NN, Ajaghaku D, Okeke HN, Ilodigwe EE, Nworu CS, Okoye FB (2014). Beta-amyrin and alpha-amyrin acetate isolated from the stem bark of Alstonia boonei display profound anti-inflammatory activity. Pharm Biol.

[B15] Ramirez-Espinosa JJ, Rios MY, Lopez-Martinez S, Lopez-Vallejo F, Medina-Franco JL, Paoli P, Camici G, Navarrete-Vazquez G, Ortiz-Andrade R, Estrada-Soto S (2011). Antidiabetic activity of some pentacyclic acid triterpenoids, role of ptp-1b: In vitro, in silico, and in vivo approaches. Eur J Med Chem.

[B16] Santos FA, Frota JT, Arruda BR, Melo TS, Silva AA, Brito GA, Chaves MH, Rao VS (2012). Antihyperglycemic and hypolipidemic effects of alpha, beta-amyrin, a triterpenoid mixture from Protium heptaphyllum in mice. Lipids Health Dis.

[B17] Sharma K, McCue P, Dunn SR (Diabetic kidney disease in the db/db mouse). 2003. Am J Physiol Renal Physiol.

[B18] Shin JA, Lee JH, Lim SY, Ha HS, Kwon HS, Park YM, Lee WC, Kang MI, Yim HW, Yoon KH, Son HY (2013). Metabolic syndrome as a predictor of type 2 diabetes, and its clinical interpretations and usefulness. J Diabetes Investig.

